# A Case Study of Acute Stimulant-induced Psychosis

**DOI:** 10.7759/cureus.4126

**Published:** 2019-02-23

**Authors:** Ashley Henning, Muhannad Kurtom, Eduardo D Espiridion

**Affiliations:** 1 Psychiatry, West Virginia School of Osteopathic Medicine, Lewisburg, USA; 2 Psychiatry, Frederick Memorial Hospital, Frederick, USA

**Keywords:** stimulant-induced psychosis, psychosis, amphetamines, methamphetamines, cocaine, nicotine, caffeine, psychotic disorders, schizophrenia, adhd

## Abstract

Psychosis resulting from stimulant overuse is commonly observed in clinical practices today. This is in large part due to the significant increase in attention-deficit/hyperactive disorder (ADHD) diagnoses in recent years, the increase in methamphetamine trafficking, and the prevalence of stimulant use in the day-to-day activities by many of those living in today’s fast-paced society. The current inability to predict those users who will experience stimulant-induced psychosis from those users who will not needs further investigation.

In this case study, we examine why one particular stimulant user experiences stimulant-induced psychosis. We give an account of a patient with an acute psychotic episode admitted to the emergency room at a local hospital. During initial evaluation, it was discovered that he had been diagnosed with ADHD one month ago and that the onset of his symptoms was likely related to an overuse of amphetamines. The patient had no personal history or family history of any psychiatric or mood disorders. He had no history of recreational drug use prior to this incident, and had no history of seizures or head trauma. After appropriate pharmacological intervention with antipsychotics and psychological intervention, the patient showed gradual improvement over the next five days of his hospitalization. After the five days, he was allowed to be discharged.

We conclude with the cautionary advice that a patient’s susceptibility of experiencing stimulant-induced psychosis should be assessed before prescribing stimulants. In cases where stimulant use is not supervised by a physician, appropriate understanding of management of stimulant-induced psychosis is of utmost importance in order to provide the very best patient education and care.

## Introduction

Stimulants are drugs that increase the body’s functions by increasing the speed of activity in the central nervous system (CNS). These drugs increase the amount of dopamine in the brain, resulting in an increase in heart rate, alertness and energy. Stimulants can be beneficial in treating medical conditions such as attention-deficit/hyperactivity disorder, narcolepsy, obesity and depression. Familiar examples of stimulants include caffeine, cocaine, amphetamines, methamphetamines and nicotine. All of these substances share common side effects due to their similar influence on the CNS. These include, but are not limited to, appetite suppression, sleep disturbances, increased blood pressure, agitation and psychosis [1]. The research here focuses on acute stimulant use resulting in psychosis.

The Diagnostic and Statistical Manual of Mental Disorders, Fifth Edition (DSM-5) criteria define substance/medication-induced psychotic disorder as the presence of delusions and/or hallucinations, with symptoms occurring soon after the intoxication or withdrawal of a substance or soon after exposure to a medication. The substance must have the potential to produce delusions or hallucinations that result in clinically significant impairment [2]. Impairment as a result of delusions or hallucinations is a prevalent side effect of stimulants, and an accepted reason why people who use them are more frequently referred to the Emergency Department or psychiatric ward than those who do not.

Acute psychosis induced by stimulants occurs within a period of four to five days after intoxication. The symptoms typically resolve with abstinence. However, recovery may be incomplete [3]. Japan, the developer of methamphetamines, has experienced major epidemics of stimulant-induced psychosis. Providentially, this has abetted further knowledge on the subject, and has led to the realization that patients suffering from this disorder can be separated into three groups. The first group includes patients experiencing transient psychosis after stimulant use that lasts for a period of four to five days following intoxication. The second group consists of patients who experience psychotic symptoms for as long as one month. The third group is comprised of patients who will not fully recover from their symptoms [3, 4]. In the Yui et al. study, 64% of patients gained full recovery from their psychosis within 10 days, 82% recovered within a month, and 18% suffered symptoms for over a month [3]. In a more recent study by Zarrabi et al., the percentage of patients who experienced persistent symptoms for over one month was 31.6%. This phenomenon has resulted in a necessary increase of beds and resources available to health care systems worldwide [4].

It should be noted that numerous studies have proven a co-morbidity of mental illness with stimulant-induced psychosis [1, 4]. However, the cause of stimulant-induced psychosis where there is no family history or prior personal history of mental illness remains unclear. More research identifying the patient population most susceptible to stimulant-induced psychosis and/or a more exact determination of the minimum dosage of each stimulant drug necessary to trigger psychotic symptoms would assist in providing better patient care through a determination of whether the benefits of the prescription outweigh the risks. Answers to these questions could also alert those patients susceptible to acute psychosis – along with their family members – to watch for changes in behavior while the patient is on the medication. Additionally, if the general population is made more aware of the risk factors associated with psychosis, it may help prevent those who are vulnerable from abusing stimulants.

## Case presentation

An 18-year-old Caucasian male with no prior psychiatric hospitalizations was sent to the local emergency room after his parents called 911; they were concerned that their son had become uncharacteristically irritable and paranoid. The family observed that their son had stopped interacting with them and had been spending long periods of time alone in his bedroom. He had also reportedly not been sleeping well and had started talking to himself. For over a month, he had not attended school at the local community college. His parents finally made the decision to call police when their son started screaming at them; the police were monitoring him at their request, soon after he threatened them with a knife. The police took him to the local emergency room for a crisis evaluation.

When the patient arrived at the emergency room, he was given intramuscular lorazepam 1 mg and haloperidol 5 mg because he attempted to strike the nursing staff and security guards. Following administration of the medication, he tried to escape from the emergency room, contending that the hospital staff was planning to kill him. The patient appeared to be internally preoccupied and his mood labile. He refused to cooperate with anyone attempting to conduct a meaningful psychiatric evaluation. He eventually slept for approximately four hours. When he awoke, he reported to the crisis worker that he had been diagnosed with attention-deficit/hyperactive disorder (ADHD) a month ago. At the time of this ADHD diagnosis he was started on 30 mg of lisdexamfetamine dimesylate to be taken every morning in order to help him focus and become less stressed over the possibility of poor school performance.

After two weeks, the provider increased his lisdexamfetamine dimesylate dosage to 60 mg every morning. The provider also started him on dextroamphetamine sulfate tablets (10 mg) that he took daily in the afternoon in order to improve his concentration and ability to study. The patient claimed that he might have taken up to three dextroamphetamine sulfate tablets over the past three days because he was worried about falling asleep, unable to adequately prepare for an examination. These were the series of events that brought him to the emergency department.

Prior to the ADHD diagnosis, the patient had no known psychiatric or substance abuse history. The urine toxicology screen taken upon admission to the emergency department was positive only for amphetamines. Other routine laboratory workups were within normal limits. He had no current history of any serious medical condition, no history of seizures or head trauma. There was no family history of psychotic or mood disorders. There were no vegetative depressive symptoms. There were no symptoms consistent with mania or hypomania. The patient denied using any illegal drug prior to this incident. He was not a victim of abuse.

The stimulant medications were discontinued by the hospital upon admission to the emergency department. The patient was treated with an atypical antipsychotic, risperidone 1 mg BID. He tolerated the medications well. He started psychotherapy sessions, and his parents visited him daily until his release five days later. On the day of discharge, there were no delusions or hallucinations reported. He was referred to the local mental health center for aftercare follow-up with a psychiatrist.

## Discussion

Prevalence of stimulant-induced psychosis

Psychosis is a symptom of a mental health illness prevalent in today’s society. As many as three in 100 people will have an episode of psychosis within their lives [5]. A study of patients admitted to the hospital with first-episode psychosis revealed that 74% of them had been diagnosed with a substance use disorder at some point in their lives [6]. This illustrates that substance use is a major cause of psychosis, a topic meriting further investigation.

A study carried out by Vallersnes et al. in 2016, ascertained the acute recreational drug toxicity commonly associated with psychosis. Psychosis occurred in 6.3% of patients admitted to the emergency department due to acute drug toxicity. Of the patients presenting with psychosis, the median age was 29; 79.3% were male and 32.8% were female. The drugs most frequently reported used were cannabis in 25.9% of cases, amphetamines in 25% and cocaine in 16.1%. More than one drug was taken in 54.3% of the cases. Amphetamine was the most frequently used drug associated with psychosis when only one agent was reported, occurring in 32.4% of the cases [7].

These results indicate that the frequency of psychosis due to acute toxicity varies widely, subject to the types of substance used. However, the greatest concern is of acute amphetamine poisoning. The difference in amphetamine poisoning occurrence between males and females is notable: 276 males presented to the emergency department, in contrast to only 72 females [7]. This may be due to the fact that males tend to participate in more risky behaviors than females [8].

In addition to the risk of inducing acute psychosis, regular use of stimulants, especially amphetamines and methamphetamines, has been found to be a major risk factor leading to the onset of chronic psychosis or schizophrenia. Yui et al. have discovered that methamphetamine users may have persisting psychotic symptoms for a duration of months to years after discontinuation of methamphetamines. Furthermore, patients with previous stimulant-induced psychosis are at greater risk of subsequent episodes [3].

Mechanism of action of stimulants

The majority of stimulants manipulate monoamine transmission. The monoamines consist of dopamine, norepinephrine and serotonin. They are relevant to the reward, motivation, temperature regulation and pain pathways. Stimulants commonly either block the reuptake or stimulate the efflux of dopamine and norepinephrine, increasing the activity within their circuitry [9].

The dopaminergic pathways altered by stimulants are the mesocortical, mesolimbic and nigrostriatal pathways. These pathways are known reward and motivational pathways. The mesocortical pathway is associated with cognitive functions, such as working memory. The mesolimbic pathway is connected with reward processing, driving emotions into actions and enmeshed with the development of behavioral patterns. The nigrostriatal pathway works to produce movement to gain reward, and leads to the development of habitual behaviors. When these pathways are altered by stimulants, they are associated with “negative” symptoms (the mesocortical pathway), “positive” symptoms (the mesolimbic pathway) and movement disorders (nigrostriatal pathway), respectively [9].

Hallucinations and delusions have been classified as “positive” symptoms. Thus, psychosis may be a result of increased activity of the mesolimbic system. The mesolimbic pathway consists of projections from the ventral tegmental area (VTA) to the nucleus accumbens, also known as the ventral striatum of the forebrain. The nucleus accumbens mediates the rewarding effects of both natural rewards and drugs of abuse that lead to the motivation to seek the reward or the liking of the reward. The study of antipsychotics has led to a better understanding of this pathway. Antipsychotics are D2 dopaminergic receptors antagonists. D2 receptors modulate transmitter release and inhibit the indirect pathway of the striatum, thereby causing more pleasure. Antipsychotics prevent the D2 receptors from inhibiting the indirect pathway, allowing the inhibitory effects of the indirect pathway to proceed [9]. This knowledge has been useful in understanding the physiology of psychosis, since antipsychotics treat positive psychotic symptoms [1, 4, 9].

It has also been hypothesized by Hsieh et al. that stimulant-induced psychosis may be the result of neurotoxicity causing damage to the cortical gamma-aminobutyric acid (GABA) interneurons. The overflow of dopamine release causes excessive enhancement of the dopaminergic pathways. This releases an abundant amount of the excitatory neurotransmitter, glutamate, into the cortex. Glutamate binds to NMDA receptors, which are highly concentrated on GABA interneurons. GABA is known as the inhibitory neurotransmitter, thus regulated concentrations of glutamate and GABA allows for proper bodily functioning. Excess glutamate can cause damage to the interneurons, causing dysregulation of the thalamocortical signals, producing psychosis [10].

The most common cause of stimulant-induced psychosis is amphetamine and methamphetamine use [7]. They inhibit dopamine reuptake into neurons by binding to dopamine transporters (DAT), resulting in an increase of the concentration of dopamine in the synaptic cleft. They also utilize the vesicular monoamine transporter 2 (VMAT2) to enter neurosecretory vesicles within the neuron. The amphetamine displaces the dopamine within the vesicles, resulting in dopamine release into the cleft [3, 10]. This prevention of dopamine reuptake with the additional release of dopamine results in an increase in concentration of dopamine in the mesolimbic and mesocortical pathways. The mesocortical pathway projects from the VTA to numerous areas of the prefrontal cortex. Projections to the dorsolateral prefrontal cortex regulate cognition and executive functioning [9].

The second most common cause of stimulant-induced psychosis is cocaine [7]. Like amphetamines, cocaine prevents the reuptake of monoamines from the synapse causing an increase in concentration of monoamines.

Two common stimulants that do not affect the dopamine pathways directly are caffeine and nicotine. Caffeine is the most widely used drug in the world. It exerts its effects by antagonizing adenosine A1 and A2A receptors, which are drivers of sleep and drowsiness. Caffeine releases the inhibitory effect of adenosine receptors on dopamine receptors, allowing an enhancement of dopamine signaling in the striatum, resulting in wakefulness and alertness. This indirect relation with the mesolimbic pathway explains the numerous case studies linked to excessive caffeine intake and psychosis [11].

Nicotine is the active chemical in tobacco products. It acts as an agonist on the nicotinic acetylcholine receptor, triggering multiple downstream effects including the increase of activity in the dopaminergic pathways. A meta-analysis by Myles et al. concluded that patients that encountered acute psychosis for the first time had either smoked for some years prior to onset or had an excess amount of tobacco ingestion when presenting the symptoms. They were more likely to have smoked than aged-matched controls [12].

Risk factors leading to vulnerability

In our case study, an excess dosage of amphetamines appears to be the underlying factor that led the patient to the emergency department. Studies by Angrist and Gershon, and Bell provide information on how amphetamines may induce psychosis in healthy individuals. In these studies, increasing doses of amphetamines were given to subjects until psychosis was triggered, usually after 100-300 mg of amphetamines. The symptoms resolved after six days [13, 14].

Amphetamines have a terminal half-life of 12-15 hours [1]. As shown in our case, amphetamines can be taken several times over the course of many days during binges. This may result in a buildup of amphetamines in the body, resulting in psychosis.

Another study correlating high doses of stimulant use to psychosis was conducted by Lucas et al. In this study, 10 mg/kg of caffeine was administered to schizophrenic patients who had been free of caffeine for six weeks. These patients charts showed an increase in the Brief Psychiatric Rating Scale (BPRS), which clinicians use to measure psychiatric symptoms such as depression, anxiety, hallucinations and unusual behavior [15]. In addition, there have been multiple case studies that show that excessive caffeine consumption during a short period of time causes psychosis and that the psychosis resolves with the stop of consumption [11].

In the case of first-episode psychosis, especially in patients with no psychiatric history, the dosage of the stimulant over a short period of time plays a role in causing psychosis. This is most likely the cause of psychosis in our case study.

The study by Lucas et al. calls attention to the relationship between schizophrenia and stimulant-induced psychosis. There have been numerous studies indicating that patients with schizophrenia, schizotypal personality traits and family history of such disorders are more prone to psychotic episodes with stimulant use [1, 3, 4, 16]. The “dopamine hypothesis of schizophrenia” attributes symptoms of schizophrenia, such as psychosis, to hyperactive dopaminergic signal transduction [16]. As explained earlier, stimulants increase the amount of dopamine in the striatum. The combination of these two factors working together to cause an overflow of dopamine in the brain makes it more likely than not that a schizophrenic patient will suffer from stimulant-induced psychosis.

It has been discovered that there are common genetic biomarkers that make a person more susceptible to amphetamine-induced psychosis and schizophrenia. One of these genes is the DAOA/G72 gene, which encodes an activator of NMDA receptors. This gene may be a reason why some people are more vulnerable to amphetamine-induced psychosis, and may explain why schizophrenics are even more vulnerable [17]. Another gene of seeming significance is the DTNBP1 dysbindin gene. Kishimoto et al. examined this gene in 197 Japanese subjects with methamphetamine-induced psychosis and 243 controls. They found that people with this gene are 2.6-7.1 times more likely to suffer from methamphetamine-induced psychosis than those who do not have the gene [18].

A phenomenon discovered from animal models, which applies to human stimulant-induced psychosis, is called “sensitization”. Sensitization occurs with repeated administration of a stimulant. Once sensitization to a stimulant develops, subsequent doses of the stimulant lead to a greater dopamine response, even after years of abstinence [19, 20]. Kazahaya et al. sighted that in previously sensitized rats, the dopamine efflux after a subsequent methamphetamine exposure increased in the striatum. They observed that an increase in dopamine occurs in animals sensitized to one stimulant and then exposed to a different one, calling this cross sensitization [19].

Similarly, Breier et al. used positron emission tomography in patients with schizophrenia, and found about twice as much amphetamine-induced dopamine in the striatum of these patients. The plasma concentration of the amphetamine was the same as the controls, signaling that the sensitivity of dopamine release into the striatum must develop through sensitization [20].

In healthy individuals, stimulant-induced psychosis appears to be the result of taking excessive amount of stimulants. However, this does not explain why some individuals experience psychosis at a certain dosage and others do not. Bramness et al. proposed that the relationship between amphetamine-induced psychosis and primary psychosis be viewed with a vulnerability stress paradigm. Individuals with low vulnerability need higher exposure to the stimulant than those who are highly vulnerable. Those individuals who are least vulnerable are those who have used amphetamines but have never experienced psychosis. Those who are most vulnerable are schizophrenic patients who become psychotic without the use of any amphetamines at all [1].

In this case study, the patient suffered from first-episode psychosis. Since the only recent change in his life was that he had been diagnosed with ADHD and had been prescribed two different kinds of amphetamines, the cause of the psychosis has been determined here to be the result of excess stimulant use.

Additionally, the vulnerability stress paradigm elucidates why we believe this healthy patient was symptomatic, while other healthy patients are generally not. The vulnerability stress paradigm implies that the more life stressors or inciting factors a person has, the lower the threshold for psychosis.

## Conclusions

In conclusion, the study urges follow-up research and documentation on first-episode patients to determine if psychosis happens again or if symptoms worsen into schizophrenia. Understanding the progression of this disease is essential to improved patient care.

For now, those who are susceptible to stimulant-induced psychosis due to medical history, a family history of mental illness, genetics, gender, and/or lifestyle must be advised and warned of the signs and symptoms of psychosis. In addition, physicians must be diligent to collect each patient’s unique history before prescribing stimulants to any patient.
